# Effect of Dietary Postbiotics Derived from *Lactiplantibacillus plantarum* and *Pediococcus lactis* on Lipid Metabolism, Blood Biochemistry, and Fecal Microbiota in Cats: A Pilot Study

**DOI:** 10.3390/vetsci13060571

**Published:** 2026-06-10

**Authors:** Huaiyu Zhang, Jintao Sun, Jinquan Wang, Xiumin Wang, Hui Tao, Zhenlong Wang, Jie Liu, Bing Han

**Affiliations:** 1 Key Laboratory of Feed Biotechnology, Ministry of Agriculture and Rural Affairs, Institute of Feed Research, Chinese Academy of Agricultural Sciences, No. 12 Zhong Guan Cun South Street, Haidian District, Beijing 100081, China; 2024240780@stu.syau.edu.cn (H.Z.);; 2 College of Animal Science and Veterinary Medicine, Shenyang Agricultural University, Shenyang 110866, China; 3 National Nanfan Research Institute, Chinese Academy of Agricultural Sciences, Sanya 572000, China

**Keywords:** cats, lipid metabolism, microbiota, postbiotics

## Abstract

Owing to their favorable stability and safety profile, postbiotics—defined as functional metabolites originating from probiotic microbes—have attracted growing interest in the field of companion animal health care. The present work sought to evaluate how postbiotics prepared from *Lactiplantibacillus plantarum* and *Pediococcus lactis* influence lipid metabolism, blood biochemistry, and the composition of fecal microbiota in felines. Eighteen clinically healthy adult cats were assigned at random to three equal-sized groups: a control treatment (*n* = 6, a basal diet), a *L. plantarum* postbiotic L-27-2 group (*n* = 6, a basal diet with 10^9^ CFU equivalent/kg/day), and a *P. lactis* L-14-1 postbiotic group (*n* = 6, a basal diet with 10^9^ CFU equivalent/kg/day). The supplementation lasted four weeks. Both postbiotics significantly lowered serum total cholesterol (TC) and triglycerides (TG) concentrations (*p* < 0.05), demonstrating a modulatory action on lipid metabolism in cats. The L-27-2 postbiotic selectively enriched beneficial taxa, notably *Bifidobacterium* (*p* < 0.05). Furthermore, fecal levels of malodorous compounds were significantly decreased by both treatments (*p* < 0.05). The results suggested both postbiotics have potential positive effects in cats. These findings are preliminary and hypothesis-generating.

## Simple Summary

Cats are popular companions and keeping them healthy is important to owners. This study tested whether postbiotics—inactive beneficial bacteria or their components—could improve the health of healthy adult cats. Eighteen cats were divided into three groups: one group ate a regular diet, while the other two ate the same diet plus one of two types of postbiotics for four weeks. The results showed that both postbiotics reduced the levels of harmful fats (cholesterol and triglycerides) in the blood. One postbiotic also increased the number of good bacteria, called Bifidobacterium, in the gut, and both postbiotics reduced bad smells in the feces. These findings suggest that postbiotics may help support healthy fat metabolism and gut health in cats. However, because the study involved only a small number of animals, more research is needed to confirm these benefits.

## 1. Introduction

Over recent decades, the domestic cat (Felis catus) has risen to become one of the world’s most widely kept companion animals. The global pet population now approaches one billion, with cats representing a large fraction [1,2]. As the emotional connection between humans and their feline companions grows stronger, owners are placing ever-greater importance on the health and well-being of their pets, viewing them as true family members [2]. As this relationship strengthens, understanding and optimizing the health of companion animals has become a priority for both owners and veterinary professionals [1].

The gastrointestinal microbiota of cats plays a fundamental role in maintaining host health, influencing nutrient metabolism, immune function, and resistance against pathogen colonization [3]. A balanced gut microbial ecosystem is essential for optimal physiological function, producing bioactive metabolites, such as short-chain fatty acids, that modulate systemic health and immune activation [4]. Conversely, dysbiosis—an imbalance in microbial community structure—has been associated with various health disorders in felines, including gastrointestinal diseases, inflammatory conditions, and metabolic disturbances [3,5]. Given the obligate carnivore nature of cats and their unique gut microbiota compared with omnivores, understanding the role of the feline gut microbiota is particularly important [6]. Disorders of lipid metabolism are clinically relevant in cats, as excessive mobilization of peripheral fat can lead to hepatic lipidosis, a potentially life-threatening condition characterized by intrahepatic accumulation of triglycerides [7].

Beyond companion animals, postbiotics have been extensively studied in livestock and laboratory animals. For instance, dietary postbiotics have been shown to improve growth performance, enhance intestinal barrier function, and modulate gut microbiota in piglets and broilers [8,9]. In rodent models, postbiotics exhibit anti-inflammatory and lipid-lowering effects [10]. These findings provide a rationale for exploring postbiotic applications in feline health.

Probiotics are live microorganisms that, when administered in adequate amounts, confer health benefits on the host [11]. Probiotics can modulate lipid metabolism mechanisms [12]. In recent years, probiotics have begun to be applied in cats. Studies have found an association between gut microbiota dysbiosis and obesity in cats. The composition of the gut microbiota in overweight and obese cats differs significantly from that in cats with healthy body weights. However, the consistency of this evidence across different studies is variable. While several reports indicate that specific probiotic strains can modulate fecal microbiota and improve certain metabolic parameters in cats, the magnitude of effect often depends on factors such as the bacterial strain used, the dosage and duration of supplementation, the age and health status of the animal, and the baseline diet [6,13]. For instance, beneficial effects on fecal consistency and immune markers have been observed with some strains but not others, and results from small-scale studies (typically *n* = 6–12 per group) may not be generalizable to broader populations. Moreover, most studies have been conducted in healthy animals, leaving the efficacy in diseased or obese cats less explored [14]. Previous research has demonstrated that *Lactiplantibacillus plantarum* L-27-2 and *Pediococcus lactis* L-14-1, isolated from feline feces, exhibit positive effects in reducing cholesterol and inhibiting pathogenic bacteria in mice [9]. Furthermore, dietary supplementation with L-27-2 and L-14-1 in cats can reduce blood lipids and inflammatory cytokines, as well as alter the microbial composition of feline feces [15]. Although probiotics are currently promoted for use in pets, their safety cannot be overlooked. Probiotics carry potential risks, including genetic instability, transmissibility, or in situ toxin production [16,17].

Postbiotics are preparations of inanimate microorganisms and/or their components that confer health benefits on the host [18]. Postbiotics have a wide range of industrial applications, particularly with innovative uses in the food, veterinary, pharmaceutical, and cosmetic industries, where they serve as functional ingredients to improve health, offering a new direction for pet health research [19]. Existing studies have shown that dietary supplementation with sodium butyrate, a postbiotic, can enhance antioxidant and anti-inflammatory capacities, improve immune function, and positively modulate gut microbiota composition in adult cats [20]. In this study, we hypothesized that dietary supplementation with these feline-origin postbiotics might be associated with improved metabolic parameters in healthy cats. Given the small sample size and the absence of baseline blood measurements, the results are intended to generate hypotheses for future confirmatory studies rather than to draw definitive conclusions.

## 2. Materials and Methods

### 2.1. Preparation of Postbiotics

In previous studies, two newly isolated strains—*Lactiplantibacillus plantarum* L-27-2 and *Pediococcus lactis* L-14-1 were obtained from the feces of healthy cats and preserved at the General Microbiology Center of the China General Microbiological Culture Collection, with deposition numbers CGMCC No. 27193 and CGMCC No. 27676 [13,15].

In this experiment, the freeze-dried stocks of L-27-2 and L-14-1 were separately inoculated into MRS liquid medium and incubated statically at 37 °C for 24 h. Single colonies were then picked and transferred into 50 mL of MRS liquid medium, followed by incubation at 37 °C for 24 h. When the optical density (OD) reached 1, the culture was inoculated at 1% (*v*/*v*) into 400 mL of fresh MRS liquid medium and incubated statically at 37 °C until the OD reached 1 again, which served as the fermentation seed culture. The fermented broth was centrifuged at 8000 rpm, 25 °C for 20 min. After discarding the supernatant, the cell pellet was resuspended in ddH_2_O and washed by repeating the centrifugation under the same conditions (8000 rpm, 25 °C, 20 min) to obtain a concentrated lactic acid bacteria paste. A sterilized lyoprotectant consisting of 10% (*w*/*v*) skim milk was added to the bacterial paste at a ratio of 1:1 (*v*/*v*) before freeze-drying, and the mixture was pre-frozen at –80 °C for 12 h, then freeze-dried at 0 Pa and –80 °C for about 24 h. The resulting freeze-dried cake was ground into a homogeneous powder. Finally, the powder was inactivated by heating in an oven at 80 °C for 30 min to obtain the postbiotic powder. After heat inactivation, the postbiotic powder was resuspended in sterile PBS and plated onto MRS agar. No colonies were observed after 48 h of aerobic and anaerobic incubation at 37 °C, confirming complete inactivation.

The dosage of postbiotics for animal administration was determined based on colony-forming unit (CFU) equivalents derived from the live bacterial cultures prior to inactivation. Specifically, live L-27-2 and L-14-1 were cultivated and harvested to achieve a concentration of 1 × 10^9^ CFU/mL, after which they were freeze-dried and heat-inactivated. This approach, commonly referred to as CFU-equivalent dosing, ensures that the administered postbiotic material originates from an equivalent amount of viable biomass, allowing for a consistent basis of comparison between the two strains.

### 2.2. Experimental Animals

The animal experiment was conducted in accordance with the guidelines of the Animal Care and Use Committee of the Institute of Feed Research, Chinese Academy of Agricultural Sciences, and was approved by the Laboratory Animal Ethics Committee and reviewed by the Institute of Feed Research, Chinese Academy of Agricultural Sciences (IFR-CAAS-20250829).

Eighteen healthy cats (3–6 years; 2–5 kg; 11 females and 7 males) were randomly assigned to three groups (CK, PL272, and PL141). Healthy status was defined as the absence of clinical signs of disease (e.g., vomiting, diarrhea, lethargy, anorexia) based on daily observation by trained personnel, no history of chronic illness, and no medication or antibiotic use within 4 weeks prior to the study. Physical examination (body condition score, coat quality, and mucosal color) was performed at enrollment. However, parasitological examinations and vaccination records were not collected; therefore, subclinical infections or vaccine-induced immune modulation may have been present. The sample size was determined based on previous similar studies [15]. The eighteen cats were randomly numbered and allocated to groups using a random number generator to avoid selection bias (Excel RAND function). All cats were not sterilized and did not use any prebiotics, probiotics, or antibiotics within 4 weeks prior to the study. The resulting sex distribution was as follows: CK group (*n* = 6; 4 females, 2 males), PL272 group (*n* = 6; 3 females, 3 males), and PL141 group (*n* = 6; 4 females, 2 males). The L-27-2 and L-14-1 groups received freeze-dried bacterial powders of L-27-2 and L-14-1, respectively, for 28 days at a dosage of 1 × 10^9^ CFU/kg/day (equivalent amount of viable bacteria). The freeze-dried powder was thoroughly mixed with the cats’ basal diet (100 g) each day before feeding. The control group was fed the basal diet without supplementation. All cats had free access to food and water. During the experiment, they were kept separately in cages (length: 162 cm; width: 62 cm; and height: 62 cm), and every day they had two hours to play.

### 2.3. Sample Collection and Fecal Score

Fecal samples were collected on day 0 and 28. Immediately after defecation, all feces were collected using clean, sterile 50 mL centrifuge tubes.

During the collection period, all fecal samples were scored every 7 days using the following scale:

1 = formed, hard, dry, small hard mass;

2 = hard, formed, dry;

3 = soft, formed, moist, shaped;

4 = soft, unformed;

5 = liquid, watery.

Blood samples were collected on day 28 via venipuncture of the saphenous vein. Blood samples were collected only on day 28 to minimize stress on the animals. We acknowledge that repeated measures would have provided stronger evidence of intra-individual changes; this limitation is addressed in the Discussion. We obtained approximately 1–1.5 mL of blood per cat. After natural clotting, the blood was allowed to settle until a clear, pale-yellow supernatant formed. Serum was then prepared by centrifugation at approximately 2000 rpm for 10 min at 4 °C.

During the experimental period, the cats were individually housed in cages and fed only a basal diet mixed with the postbiotic powder; the basal diet was a commercial, grain-free, complete dry feline diet formulated for adult cats (Golden Premium Adult Cat Food, Chicken Recipe, produced by Haozhuren, China). All cats were fed ad libitum with free access to water, and no antibiotics were administered throughout the trial.

### 2.4. Blood Biochemical Tests

Serum biochemical parameters were analyzed using a fully automated biochemical analyzer (MNCHIP, Tianjin, China). The panel included total bile acids (TBAs), total cholesterol (TC), and triglycerides (TGs) as primary outcomes [21]. In addition, a comprehensive set of other parameters was measured to evaluate overall health and metabolic status, including total protein, albumin, globulin, albumin/globulin ratio, total bilirubin, alanine aminotransferase (ALT), gamma-glutamyl transferase (GGT), alkaline phosphatase (ALP), creatine kinase, amylase, creatinine, urea nitrogen, BUN/creatinine ratio, and inorganic phosphorus. All assays were performed according to the manufacturer’s protocols, and the analyzer was calibrated daily with standard controls.

### 2.5. Measurement of Fecal Skatole and Indole Levels

The skatole and indole levels of all the fecal samples were measured using ELISA kits (Jiangsu Enzyme Immunoassay Industrial Co., Ltd., Yancheng, China) [15,21].

### 2.6. 16S rRNA Gene Amplicon Sequencing and Bioinformatics Analysis

Microbial DNA was extracted from feline fecal samples using the E.Z.N.A.™ Mag-Bind Soil DNA Kit (Omega Bio-tek, Norcross, GA, USA) according to the manufacturer’s instructions. The V3–V4 hypervariable region of the bacterial 16S rRNA gene was amplified using the universal primers 338F (5′-ACTCCTACGGGAGGCAGCAG-3′) and 806R (5′-GGACTACHVGGGTWTCTAAT-3′). PCR reactions were performed in a total volume of 30 µL containing 2 µL of DNA template (10 ng/µL), 1 µL of each primer (10 µM), and 15 µL of 2× Hieff Robust PCR Master Mix (Yeasen, Shanghai, China). The thermal cycling conditions were as follows: initial denaturation at 95 °C for 3 min; 5 cycles of denaturation at 95 °C for 30 s, annealing at 45 °C for 30 s, and extension at 72 °C for 30 s; followed by 20 cycles of denaturation at 95 °C for 30 s, annealing at 55 °C for 30 s, and extension at 72 °C for 30 s; with a final extension at 72 °C for 5 min. All PCR reactions were performed in triplicate and pooled. Amplicons were verified by 2% agarose gel electrophoresis and purified using Hieff NGS™ DNA Selection Beads (Yeasen, China). PCR was performed in a Bio-Rad T100 thermal cycler (Bio-Rad, Hercules, CA, USA). The expected amplicon size was approximately 460 bp. No-template controls (NTCs) were included in each PCR run to rule out contamination. Libraries were constructed using universal Illumina adapters and indices, then sequenced on an Illumina MiSeq platform (2 × 300 bp paired-end) at Sangon Biotech (Shanghai, China).

Initial processing of raw reads involved removal of adapter and primer sequences with cutadapt (v1.18). Overlapping paired-end reads were then assembled into longer sequences using PEAR (v0.9.8) under default settings. For quality control, we applied PRINSEQ (v0.20.4) with a 10-nucleotide sliding window: any read whose average Phred quality dropped below 20 within a window was trimmed from that point onward; reads containing ambiguous nucleotides or shorter than 50 bases after trimming were discarded.

Using USEARCH (v11.0.667), we clustered the high-quality reads into operational taxonomic units (OTUs) at a 97% sequence identity threshold. The clustering process included chimera detection and removal; moreover, any OTU supported by only a single read (singleton) was eliminated from further analysis. Taxonomic assignment was performed by comparing each OTU’s representative sequence against the SILVA reference database (release 138) using the RDP classifier (v2.12), accepting assignments with a confidence score of ≥0.8.

Alpha-diversity metrics, including Shannon, Simpson, and Chao1 indices, were computed with Mothur (v1.43.0). For beta-diversity analysis, we performed principal coordinate analysis (PCoA) based on Bray–Curtis distances and tested the statistical significance of group differences using permutational multivariate analysis of variance (PERMANOVA) with the ‘adonis2’ routine in the R package ‘vegan’ (v2.5–6). The permutation test was run with 999 iterations. Differentially abundant microbial features between groups were identified using linear discriminant analysis effect size (LEfSe) on the Huttenhower lab’s online platform. The analysis used a Kruskal–Wallis test (alpha = 0.05), followed by pairwise Wilcoxon tests (alpha = 0.05) for subgroups, and an LDA score cutoff of 2.0 (on a log10 scale) to determine effect size [15].

### 2.7. Statistical Analysis

Data for blood parameters and selected fecal indicators (e.g., sIgA and indole) were analyzed by one-way ANOVA followed by Tukey’s multiple-range test. Data are presented as means ± standard error (SE) based on Tukey’s test, and significance was considered when *p* < 0.05. These statistical analyses were performed using GraphPad Prism 8.0.2 software.

It should be noted that *p* < 0.05 was considered statistically significant. The following *p*-value notations were used: * *p* < 0.05, ** *p* < 0.01.

## 3. Results

### 3.1. Body Weight and Feed Intake

From Table 1, it was concluded that there was no significant difference in the body weight and daily feed intake among different treatment groups (*p* > 0.05).

### 3.2. Blood Biochemistry Indicators

**Figure 1:**
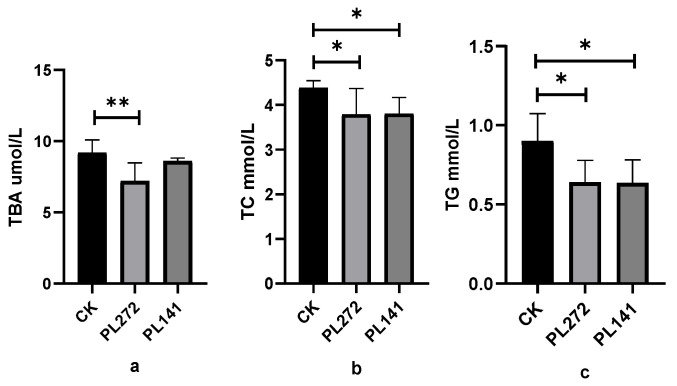
Lipid metabolism indicators. (**a**) Blood TBA levels. (**b**) Blood TC levels. (**c**) Blood TG levels. Note: * *p* < 0.05, ** *p* < 0.01.

As shown in Figure 1, both the PL141 and PL272 groups significantly reduced TC and TG levels in blood (*p* < 0.05), while TBA was only significantly reduced in the PL272 group (*p* < 0.01).

The other serum biochemical profiles are presented in Table 2. In general, most parameters remained within reference ranges for healthy cats. Notably, supplementation with PL272 and PL141 significantly decreased serum alkaline phosphatase (ALP) activity (*p* = 0.042) compared with the CK group.

### 3.3. Skatole and Indole

**Figure 2:**
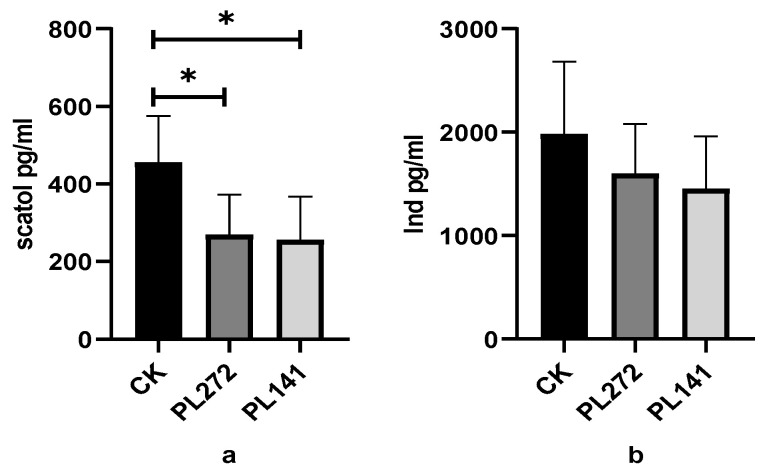
Fecal levels of skatole and indole in cats after 28 days of supplementation. (**a**) Fecal skatole content. (**b**) Fecal indole content. * *p* < 0.05.

As shown in Figure 2a, compared to the CK group, PL272 and PL141 could both significantly decrease the fecal skatole contents (*p* < 0.05), while there was no significance in indole content (Figure 2b) (*p* > 0.05).

Fecal scores were recorded weekly, as shown in the Methods Section. As shown in Table 3, no significant differences were observed among groups at baseline (Day 0, *p* = 0.152). Over the 28-day trial, all groups showed a slight increase in fecal scores (Day 28, *p* = 0.233), but the result was not statistically significant.

### 3.4. Fecal Microbiota

**Figure 3:**
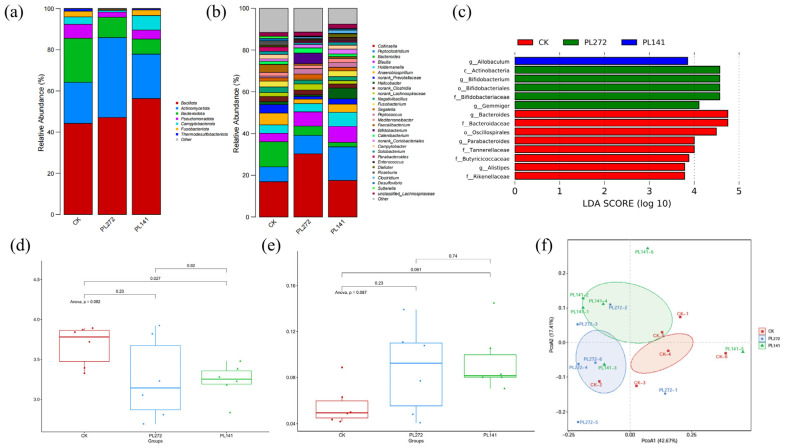
The diversity and relative abundance of fecal microbiota. (**a**) The abundance at the phylum level. (**b**) The abundance at the genus level. (**c**) Exploratory LEfSe analysis of differentially abundant taxa among groups. (**d**) α-diversity analysis (Shannon index). (**e**) α-diversity analysis (Simpson index). (**f**) Principal coordinate analysis (PCoA) at the phylum level.

Microbial community profiling was conducted by sequencing fecal samples from cats. Community diversity was evaluated through both α- and β-diversity analyses. For α-diversity, the Shannon and Simpson indices indicated a significant difference between the CK and PL141 groups (Figure 3d,e, Table 4). For β-diversity, principal coordinate analysis (PCoA) was applied to visualize compositional differences among samples, with the phylum-level PCoA plot shown in Figure 3f, which showed that PCoA1 and PCoA2 explained 42.67% and 17.41% of the total variation, respectively, with a cumulative explanatory power of 60.08% (*p* = 0.045), which could effectively reflect the differences in different groups.

As shown in Figure 3a, at the phylum level, the relative abundance of Bacillota was higher in the PL141 group than the CK group. These relative abundance patterns suggested a shift in community composition but did not demonstrate causal promotion of proliferation. The abundance of Actinomycetota showed an increasing trend in the PL272 group compared with the CK group, suggesting that the two postbiotics may exert a similar regulatory direction on gut microbiota composition.

At the genus level (Figure 3b,c), the relative abundance of *Gemmiger* and *Bifidobacterium* was increased in the PL272 group, and the relative abundance of *Allobaculum* was increased in PL141 compared to the CK group separately.

## 4. Discussion

Postbiotics offer practical advantages in storage stability and absence of live-cell safety concerns, although direct comparative safety studies with live probiotics are lacking [18]. Accordingly, in this study, we utilized strains isolated from feline feces to prepare postbiotics for experimentation.

Both L-27-2 and L-14-1 postbiotics significantly reduced total bile acids (TBAs), total cholesterol (TC), and triglycerides (TGs) in healthy adult cats (Figure 1a–c). Body weight and feed intake did not differ among groups (Table 1), indicating that the lipid-lowering effects were not due to altered energy intake. These findings are consistent with our previous report that live *Lactiplantibacillus plantarum* lowers blood lipids in hyperlipidemic rats and cats [13,14,15], suggesting that the inactivated preparations retain lipid-regulating components. Similar lipid-lowering effects have been reported for *Lactobacillus rhamnosus* GG in obese mice [22]. Moreover, in piglets, yeast-derived postbiotics have been shown to improve lipid profiles [23], and in broilers, postbiotics reduced abdominal fat deposition [24]. These cross-species comparisons suggest that the observed metabolic benefits in cats are consistent with broader postbiotic functions. Serum TBA reflects a balance of hepatic synthesis, reabsorption, and excretion [25]; therefore, the reason for TBA decrease could result from enhanced fecal elimination or altered reabsorption. The increased abundance of *Bifidobacterium* and *Parabacteroides*, both associated with bile acid metabolism, may contribute to improving lipid profiles [26]. However, because we did not measure fecal bile acids or bile salt hydrolase activity, the mechanism remained speculative.

Fecal skatole, a major putrefactive product of tryptophan metabolism, was significantly reduced by both postbiotics (Figure 2a), whereas indole levels remained unchanged. Skatole is a key contributor to fecal malodor in carnivores, and its decrease indicates attenuated protein fermentation and an improved intestinal microecological environment. A previous cat study also reported similar reductions in microbial-derived putrefactive compounds [27]. The reduction in skatole may result from inhibition of specific skatole-producing bacteria or from competitive suppression of abnormal protein fermentation by beneficial bacteria. Numerically improved fecal scores (Table 3) further support a beneficial effect on intestinal health, although the differences were not statistically significant.

Feeding L-27-2 and L-14-1 postbiotics significantly reshaped the fecal microbiota of healthy adult cats (Figure 3). Such changes in microbial composition are known to influence host metabolism and immune status [28]. At the phylum level, PL141 increased the relative abundance of Bacillota, while PL272 increased the relative abundance of Actinomycetota. At the genus level, the PL272 group increased the abundance of *Bifidobacterium* and *Gemmiger*, whereas the PL141 group promoted the abundance of *Allobaculum*. These genera are known producers of short-chain fatty acids (SCFAs) in other studies [29]; SCFAs are crucial for gut health and have systemic metabolic effects [28]. *Bifidobacterium* has been reported to participate in cholesterol reduction via bile salt hydrolase [30]. The enrichment of Bifidobacterium in the L-27-2 group aligns with findings in dogs and humans, where postbiotics and prebiotics selectively promote beneficial commensals [31]. *Parabacteroides* species exert therapeutic effects against metabolic disorders through SCFA secretion and bile acid transformation [32]. In cats, supplementation with a *Bifidobacterium*-derived postbiotic has been shown to modulate fecal metabolites and systemic markers, suggesting a potential for similar effects in our study [33]. Moreover, a specific *Bifidobacterium pseudolongum*-derived bile acid has been demonstrated to attenuate colitis in mice by modulating the cGMP-PKG-mTORC1 pathway, indicating that *Bifidobacteria* can influence host metabolism via bile acid signaling [34]. Cats are obligate carnivores, and their gut microbiota differs fundamentally from that of omnivores, typically dominated by protein-fermenting bacteria [6,35]. The successful modulation of feline microbiota by autochthonous postbiotics is noteworthy, suggesting that inactivated cellular components of host-derived symbionts can act as ecological modulators. Nevertheless, the OTU method was used to analyze the microbiota in this test. Compared with ASV, OTU clustering may merge some closely related sequences and reduce the taxonomic resolution to a certain extent, which may lead to a slight overestimation of microbial community diversity. In the future, we will use the ASV method to analyze the microbiota.

As this is a pilot study, there are several limitations. First, the study population was small and heterogeneous in age (3–6 years) and sex distribution. Although sex was included as a covariate and no significant sex-related differences were detected, the small sample size limits the power to fully control for these factors. Second, we did not measure lipoprotein subfractions (HDL, LDL, and VLDL) and baseline blood levels on day 0, which are clinically more informative for evaluating obesity-related dyslipidemia. Third, the duration of the test was only 28 days, and it was a pilot study and maybe too short for blood results. Given these limitations, the findings should be considered hypothesis-generating and exploratory. Future studies with larger sample sizes and full lipoprotein profiling are needed to validate our observations.

## 5. Conclusions

In this exploratory study, supplementation with postbiotics derived from *Lactiplantibacillus plantarum* L-27-2 and *Pediococcus lactis* L-14-1 could lower serum TC and TG in cats, as well as shift fecal microbiota composition in cats, which shows postbiotics can possibly be beneficial for the health of cats.

## Figures and Tables

**Table 1:** Body weight and feed intake parameters of cats in different groups.

Group	Initial Body Weight (kg)	Final Body Weight (kg)	Body Weight Change (kg)	Daily Feed Intake (g/d)	Feed Intake/Body Weight (g/kg/d)
CK	3.35 ± 0.38	3.42 ± 0.38	+0.07 ± 0.06	99.9 ± 6.1	29.1 ± 3.3
PL272	4.08 ± 1.07	4.04 ± 1.12	−0.04 ± 0.21	100.5 ± 2.5	25.7 ± 6.7
PL141	3.69 ± 1.05	3.76 ± 1.13	+0.07 ± 0.17	104.3 ± 3.9	30.2 ± 9.5
*p*-value	0.442	0.522	0.476	0.092	0.404

**Table 2:** Serum biochemical parameters of cats in different groups (CK, PL272, and PL141).

Parameter (Unit)	CK (*n* = 6)	PL272 (*n* = 6)	PL141 (*n* = 6)	Reference Range	*p*-Value
Albumin (g/L)	35.9 ± 2.9	35.0 ± 4.1	30.2 ± 3.1	22–45	0.025
Globulin (g/L)	49.9 ± 8.9	56.7 ± 16.4	53.3 ± 10.3	15–57	0.648
Inorganic phosphorus (mmol/L)	2.21 ± 0.25	1.49 ± 0.34	1.75 ± 0.12	1–2.5	<0.001
Albumin/Globulin ratio	0.73 ± 0.11	0.64 ± 0.17	0.60 ± 0.09	—	0.243
Total bilirubin (μmol/L)	6.00 ± 1.45	6.20 ± 1.15	5.73 ± 1.35	2–15	0.786
ALT (U/L)	65.0 ± 39.5	54.3 ± 8.1	70.0 ± 59.9	8.2–123	0.812
GGT (U/L)	1.42 ± 0.55	1.30 ± 0.73	1.37 ± 0.38	0–2	0.939
ALP (U/L)	35.0 ± 7.2	25.5 ± 9.0	23.2 ± 6.9	10–90	0.042
Creatine kinase (U/L)	182 ± 54	198 ± 115	143 ± 45	69–214	0.476
Amylase (U/L)	1722 ± 218	1912 ± 649	1590 ± 343	400–3000	0.453
Creatinine (μmol/L)	119.5 ± 15.0	115.7 ± 15.9	119.8 ± 14.3	89–207	0.860
Urea nitrogen (mmol/L)	8.71 ± 1.06	8.04 ± 1.02	8.06 ± 0.88	6.6–11.3	0.434
BUN/Creatinine ratio	18.8 ± 2.1	16.8 ± 0.8	16.8 ± 1.5	—	0.059

**Table 3:** Fecal scores of cats in different groups over the 28-day trial.

Group	Day 0 (8.28)	Day 7 (9.4)	Day 14 (9.11)	Day 21 (9.18)	Day 28 (9.25)
CK	1.92 ± 0.49	2.00 ± 0.55	1.67 ± 0.61	2.67 ± 0.41	2.67 ± 0.26
PL272	1.50 ± 0.55	2.00 ± 0.63	1.83 ± 0.41	2.33 ± 0.52	2.83 ± 0.26
PL141	1.33 ± 0.52	1.83 ± 0.41	1.42 ± 0.58	2.25 ± 0.27	2.92 ± 0.20
*p*-value	0.152	0.783	0.321	0.084	0.233

**Table 4:** α-diversity indices.

Indices	CK	PL272	PL141	*p* Value
Shannon index	3.673	3.230	3.255	0.082
Simpson index	0.055	0.093	0.087	0.086

## Data Availability

The original contributions presented in this study are included in the article. Further inquiries can be directed to the corresponding author.
